# Pediatric Atypical Melanocytic Proliferations: Single-Site Retrospective Cohort Assessment of Treatment and Long-Term Follow-Up

**DOI:** 10.3390/cancers15245804

**Published:** 2023-12-12

**Authors:** Emily Hatheway Marshall, Gabriella Alvarez, Bangchen Wang, Jennifer Crimmins, Michelle M. Schneider, M. Angelica Selim, Rami N. Al-Rohil

**Affiliations:** 1 Department of Pathology, Mass General Brigham, Boston, MA 02130, USA; ehathewaymarshall@mgb.org; 2 Department of Internal Medicine, The University of Texas Health Sciences Center at Houston, Houston, TX 77030, USA; gabriella.v.alvarez@uth.tmc.edu; 3 Department of Pathology, Duke University Medical Center, Durham, NC 27710, USA; bangchen.wang@duke.edu (B.W.); jennifer.crimmins@duke.edu (J.C.); angelica.selim@duke.edu (M.A.S.)

**Keywords:** pediatric melanoma, Spitz tumor, pigmented epithelioid melanocytoma, deep penetrating nevus

## Abstract

**Simple Summary:**

Pediatric skin lesions of melanocytes are understudied, with limited data regarding long-term survival and treatment methods. Melanocytes are cells that produce melanin and cause pigmentation in the skin. The authors sought to add to the literature by looking at all cases of atypical melanocytic proliferations that were seen at our institution over a twenty-year period; there were 166 such cases. With these data, we compared the findings, such as follow-up time, location of disease, and treatment method, to other studies on childhood atypical lesions of melanocytes. Our findings were similar to prior studies in terms of location of primary disease and overall high survival rate. There were exceptions in positive lymph node rate for pediatric melanoma, a lower excision rate for atypical Spitz tumors, and a patient who died from atypical Spitz melanoma.

**Abstract:**

Atypical and malignant cutaneous tumors are understudied in the pediatric population, with limited data on long-term follow-up. This study examines pediatric (0–18 years) atypical melanocytic proliferations over a twenty-year period (January 2002–December2022) using the EPIC SlicerDicer at our institution. Over a twenty-year period, there were 55 cases of pediatric melanoma (53 patients). The median follow-up time was 8 years, 11 months. A proportion of 96% were treated with wide local excision (WLE), and 47% had a sentinel lymph node biopsy (SLNB) (35% positive rate). There were 101 atypical Spitz tumor cases (85% atypical Spitz tumors, 15% Spitz melanoma), with a median follow-up duration of 9 years. A proportion of 77% were treated with WLE (with one patient dying of metastatic disease). There were 10 cases of atypical melanocytic proliferations not otherwise specified, including 5 pigmented epithelioid melanocytomas (PEM), 4 deep-penetrating nevi, and 1 atypical cellular blue nevus. This study adds to the growing body of knowledge on pediatric atypical cutaneous melanocytic proliferations, aligning with many described characteristics such as disease location and overall survival rates, with distinct exceptions (higher melanoma positive SLNB rate, lower atypical Spitz tumor WLE rate, and a case of fatal metastatic atypical Spitz tumor).

## 1. Introduction

Atypical or malignant cutaneous tumors are rare in the pediatric population. Melanocytic tumors, in particular, are challenging to diagnose in children because they do not necessarily present as they would in adults (i.e., melanocytic tumors secondary to UV-light damage), and many entities are challenging to clinically and histologically differentiate from melanoma [1,2,3,4,5,6,7,8,9]. Given the relative rarity of these entities, more studies are necessary to fully characterize their prognostic potential. 

Pediatric cutaneous melanomas are extremely uncommon and are estimated to occur between 1 and 11 times per 1 million children globally [5,6,10,11]. A correlative increase in incidence in age is reported: 1 per 1 million reported in children under ten years of age, and about 10 per 1 million in patients aged ten years or older [10]. Pediatric melanoma is poorly understood given its rarity and there are limited studies on it, with a particular lack in studies concerning treatment and long-term follow-up. Like adults, children with head and neck melanoma are reported to have worse prognoses and more lymph node involvement than other locations on the body [10]. In a cohort of non-head-and-neck pediatric melanomas, trunk lesions have a higher mortality than those in the extremities [5]. Management of these rare entities in the pediatric population relies primarily on retrospective data to further elucidate these disease processes, with a consensus on wide local excision (WLE) and possible sentinel lymph node biopsy (SLNB) [3,9,10,12,13,14]. Despite the paucity of data, the overall 5-year survival for patients with pediatric melanoma has been reported as 87–95% in a review article [4]; additional studies have shown an 85% [5] survival rate in a cohort of 78 cases in British Columbia, and 37 cases [3] have reported a 67.7% survival rate in Australia and New Zealand. Molecular analysis has described BRAF mutations in the adult melanoma population, and a study highlighted BRAF testing and positivity in a pediatric melanoma cohort [3,15]. Additionally, CDKN2A mutations have been described [16]. Continued contributions to the literature will help further knowledge on pediatric melanoma treatment, molecular alterations, and prognosis. 

Another category of pediatric atypical melanocytic tumors is atypical Spitz proliferation. These lesions can clinically resemble other benign cutaneous findings clinically, and their histological assessment is complex, making their prompt diagnosis challenging [2,7,17,18]. Spitz proliferations range from benign Spitz nevi to atypical Spitz tumors and Spitz melanoma [17,19,20]. The incidence of Spitz nevi is estimated to be 1–2% of all melanocytic lesions diagnosed across all age groups [20]. The incidences of atypical Spitz tumors and Spitz melanomas are not known, but both are less commonly seen than Spitz nevi [20]. Atypical Spitz tumors, in particular, are described as having minimal lethal potential, with a reported mortality rate of less than 5% in the pediatric population [2]. Spitz melanoma is extremely rare; a recent review by Yeh et al. suggests that, although there are insufficient data for definitive prognostic conclusions, cohort studies suggest that the outcomes for patients with Spitz melanomas are more favorable than a conventional melanoma of a similar thickness in the pediatric population [17]. Atypical Spitz tumors and Spitz melanomas do not currently a have consensus on the utility of SNLB, but WLE is the recommended treatment in the pediatric population [2,17,20]. Atypical Spitz tumors, primarily in adults but also in the limited pediatric literature, have been described to harbor a variety of genetic mutations and fusions, including BRAF, HRAS, CDKN2A, and TERT-p, with TERT mutations suggesting a worse prognosis in the pediatric population [19,21]. Atypical Spitz tumors and Spitz melanomas typically have better prognoses compared to melanoma, although they still may require aggressive treatment [17,19,22]. More data are necessary to characterize these lesions fully. In a retrospective study of 52 pediatric melanomas by Carrera et al. [6], they concluded that pediatric melanomas can be classified as Spitzoid and Non-Spitzoid. In Non-Spitzoid cases, melanomas presented at a mean age of 16.3 and were associated with a high-risk phenotype and arising within a preexisting nevus. Spitzoid melanomas were diagnosed at a mean age of 12.5 and were mostly de novo lesions. It is worth noting in this study that less than 25% of pediatric melanomas fulfilled the modified clinical ABCD criteria; however, 40% of Spitzoid melanomas did. Dermoscopically, Spitzoid melanomas revealed atypical vascular patterns with shiny-white lines or atypical pigmented Spitzoid pattern [6].

Pigmented epithelioid melanocytomas (PEMs) and deep-penetrating nevi (DPN) are two uncommon lesions that can be challenging to differentiate both clinically and histologically from melanoma [1,23]. A 10-year retrospective review from a single institution identified only nine cases of PEM across all age groups; this cohort highlighted a high SLNB-positive rate [23] that was consistent with the limited literature supporting these tumors’ high regional, but not systemic, metastatic potential [24]. DPNs, particularly in the pediatric population, have been found to be challenging to differentiate from melanoma [1]. In a systematic review by Cosgarea et al. 2020, DPNs (adult and pediatric cases) were seen mostly as benign lesions with low metastatic potential [1]. This is consistent with more recent literature, which suggests low metastatic potential and favorable outcomes for patients with DPN [25]. There is a lack of pediatric-specific literature that thoroughly describes the lesions in both PEMs and DPNs.

This study aims to contribute to the literature regarding pediatric melanocytic tumors. Given the limited literature on these cases’ treatment and long-term follow-up, sharing data from our institution may help broaden the understanding of pediatric cutaneous tumors. 

## 2. Materials and Methods

A single-site retrospective cohort design was used to assess the treatment and long-term follow-up for the pediatric population with atypical melanocytic lesions at our institution. IRB Review was approved (Pro00111396).

A chart review was performed from 1 January 2002 to 31 December 2022. Patients with melanoma were identified through the SlicerDicer function in EPIC (sorting age at collection 0–18 years, and “melanoma” in the testing context). To identify all patients with atypical Spitz tumors, the SlicerDicer function was used (sorting age at collection 0–18, and “Spitz” and/or “atypical Spitz” in the testing context). Inclusion criteria included a diagnosis of melanoma (diagnosis made or slide confirmation/consult at the institution) or a diagnosis of atypical Spitz tumor (diagnosis made or slide confirmation at the institution). Exclusion criteria included the following: diagnosis made elsewhere; no pathology follow-up at our institution (no access to full pathology report); lesions favored to be Spitz nevi or benign in nature by clinical correlation when the pathologic diagnosis included a differential. Patient’s current age, age at diagnosis, biological sex at diagnosis, pathologic diagnosis (with staging), the occurrence of SLNB, metastatic disease (SLN and systemic), treatment, genetic/molecular studies, and most recent follow-up data were collected. For this study, we defined lost to follow-up (LTFU) as less than six months post-diagnosis follow-up. Follow-up duration was calculated from the date of diagnosis to the most recent follow-up date or the date of death for those patients who are deceased. The results were tabulated, and descriptive statistical analysis was performed.

## 3. Results

### 3.1. Melanoma

Fifty-three pediatric patients with melanoma were identified (for a total of fifty-five cases of pediatric melanoma during the study timeframe). Characteristics are summarized in Table 1. Six patients had more than one melanoma documented (two patients had their second pediatric melanoma diagnosed during the timeframe of this study, and four were adults at the time of subsequent melanoma diagnoses). 

The age range at diagnosis overall was 4 months–18 years, with a median of 16 years and a mean of 14 years and 11 months. There were 26 male (49%) and 27 female (51%) patients represented in this case series. The anatomic locations were the following: trunk (53%, 29/55), head and neck (22%, 12/55), legs (15%, 8/55), arms/hands (5%, 3/55), and feet (5%, 3/55). A proportion of 96% (53/55) of cases were treated with WLE (1 patient had WLE scheduled but not performed, and 1 patient was a slide consult with no follow-up data). A proportion of 49% (26/53) of patients had SLNB along with WLE. Of those patients, 35% (9/26) had metastatic melanoma in the sentinel lymph node. A proportion of 7% of patients (4/55) had systemic metastatic melanoma. Overall, the median follow-up time for all cases of melanoma was 8 years, 11 months, with a mean follow-up of 9 years, 2 months. A proportion of 75% (40/53) of patients were alive without disease at the time of the study. Eight patients (15%, 8/53) were lost to follow-up (defined as <6 months of follow-up data available); of these patients, two were diagnosed in 2022, and one was diagnosed within six months of the end of the date range of the study. A proportion of 8% (4/53) died of disease at the time of analysis.

There were 12 cases of melanoma in situ (MMIS) and 42 invasive melanoma. For melanoma in situ (one patient was LTFU, N = 11), the range in follow-up duration was 7 months–21 years, 2 months with a median of 14 years, 6 months and a mean of 11 years, 10 months. All of the cases of MMIS at follow-up were alive without disease, except one patient who was deceased without disease at the time of the study due to acute lymphoblastic leukemia (ALL) (1 year, 3 months after diagnosis of MMIS).

In invasive melanoma (N = 35, 7 LTFU), follow-up duration ranged from 6 months to 21 years, 2 months, with a mean of 8 years, 9 months, and a median of 7 years, 7 months. The median Breslow thickness was 1.1 mm, with a mean of 1.68 mm for all cases of invasive melanoma. Of the 22 patients with documented Breslow thickness of 1 mm or greater, all but one (95%, 21/22) had SLNB.

Systemic metastatic melanoma was documented in four cases of invasive melanoma. The average Breslow thickness for these patients was 5.23 mm (median of 3.45 mm). Locations of metastasis were the following: brain (including leptomeningeal spread and ventricular ependymal lesions), lungs, epidural, mediastinal nodes, bone (including spine, pelvis, femur), chest wall, and local spread (occipital area, axillary, intraparotid lymph nodes). A proportion of 100% received WLE and SLNB, with 100% positive rate (4/4). A proportion of 100% of these patients received chemotherapy (including interferon, ipilimumab/nivolumab, infliximab, dabrafenib, trametinib, and vemurafenib). A proportion of 100% of the patients with systemic metastatic disease tested positive for BRAF V600E mutation (4/4). A proportion of 75% (3/4) of these patients were deceased with disease at the time of the study. 

For survival, four patients were deceased with disease at the time of the study. In addition to three patients with widely metastatic disease, one patient with a melanoma of the spindle cell type arising in the background of a congenital lesion; they died of disease. The range of time from diagnosis to death for these patients was 1 year, 4 months–7 years, 7 months, with a median duration of 4 years, 4 months, and a mean duration of 4 years, 5 months. One patient was deceased without disease at the time of the study due to acute lymphoblastic leukemia (ALL) (1 year, 3 months after diagnosis of MMIS).

Nine patients (17%, 9/53) received systemic chemotherapy treatment, including infliximab, trametinib, interferon, ipilimumab, nivolumab (PD1 inhibitor), pembrolizumab (PD1 inhibitor), vemurafenib (anti-BRAF), and dabrafenib (anti-BRAF). This relatively low percentage may be due in part to the diagnosis and treatment of many cases in this study being performed at a time prior to the era of immunotherapy as a treatment methodology for melanoma. 

Five patients were positive for the BRAF V600E mutation. Of these, 80% (4/5) received anti-BRAF chemotherapy. The patient that did not receive anti-BRAF chemotherapy had a positive sentinel lymph node but no systemic metastasis at 4 years of follow-up, and did not receive any systemic chemotherapy treatment. A proportion of 60% (3/5) of the patients with BRAF V600E mutations were deceased with disease at the time of the study, with 40% (2/5) alive without disease.

### 3.2. Atypical Spitz Proliferations

There were 101 cases of atypical Spitz proliferations, including atypical Spitz tumors and Spitz melanoma. Characteristics of the cohort are summarized in Table 2. The age range at diagnosis was 7 months–18 years. The median age at diagnosis overall was 7 years (mean of 8 years, 5 months). The sex at diagnosis was 47% (47/101) male and 53% (54/101) female. The median follow-up duration was 9 years (mean of 8 years, 10 months). For location, legs were the most common (30%, 30/101), followed by arms/hands (25%, 25/101), head and neck (24%, 24/101), trunk (18%, 18/101), and feet (4%, 4/101). For treatment, 77% (78/101) of patients had a WLE, and of those, 19% (15/78) had an SLNB. The positive rate for SLNB was 20% (3/15). One patient had systemic metastatic disease and died of disease (1%, 1/101). This patient is further discussed below. A proportion of 74% of patients were alive without disease at the time of the study (75/101). A proportion of 20% were LTFU (20/101) and 5% were alive without documentation of excision/clear margins to be categorized as without disease (that said, there were no recurrences or progression of disease in the chart for these patients). 

There were 15 cases of Spitz melanoma (15%, 15/101). The age range of diagnosis was 4–16 with a median of 11 years and a mean of 10 years, 6 months. The median (mean) follow-up duration was 8 years 7 months (8 years 6 months). There were 8 males (53%) and 7 females (47%). The locations were the following: trunk (33%, 5/15), head and neck (27%, 4/15), legs (20%, 3/15), and arms (20%, 3/15). The average Breslow thickness was 2.54 mm. The WLE rate was 93%; all cases recommended WLE, but one patient did not have documentation of WLE (punch biopsy), so 14/15 patients received the recommended WLE. A proportion of 80% of patients had an SLNB (12/15), with a 25% (3/12) positive rate. One patient had systemic metastatic disease (1%, 1/101) of all cases; 7% (1/15) of Spitz melanomas). This patient had documented metastatic disease (regional LN, 3 cm extra-nodal mass, and CT suspicious for multiple lung metastases). It is noteworthy that this patient had a lesion removed three years prior that was diagnosed as an atypical Spitz nevus with negative margins at the same site of this case of Spitz melanoma. Molecular analysis showed a wild-type (WT) BRAF. Ipilimumab was discussed, but the patient did not receive chemotherapy prior to death. A proportion of 80% of the patients diagnosed with Spitz melanoma were alive without disease at the time of the study (12/15), 7% (1/15) were LTFU, 7% (1/15) had long-term follow-up but no documented excision, and 7% (1/15) were deceased with disease at the time of the study. 

### 3.3. Atypical Melanocytic Tumors, Not Otherwise Specified

Ten patients fell into the category of melanocytic tumors, not otherwise specified. Of these lesions, five were diagnoses of PEMs, four were atypical DPN, and one was an atypical cellular blue nevus. Characteristics are summarized in Table 3.

For the PEM cases, the age range at diagnosis was 3–16 years old, with a median of 10 and mean of 9 years, 7 months. A proportion of 80% of patients were male, and 20% were female (4/5,1/5). The locations of the lesions were as follows: head and neck (40%, 2/5), trunk (40%, 2/5), and leg (20%, 1/5). A proportion of 100% of patients had a WLE, and 80% had SNLB. Of those, 50% (2/4) had positive SNLB. One patient had regional metastatic disease and received interferon chemotherapy. The follow-up time range was 3 years, 8 months–12 years, 8 months, with a median of 6 years, 11 months and a mean of 8 years, 2 months. A proportion of 100% of patients are alive without disease at the time of the study.

There were four cases of atypical deep-penetrating nevi, with an age range at diagnosis of 2–18, with a median of 14 years 6 months and a mean of 12 years 3 months. A proportion of 75% of patients were male, and 25% female. A proportion of 50% of cases were of the head and neck, and 50% were of the trunk. A proportion of 50% of patients had a WLE, and 50% had either a shave or punch biopsy. A proportion of 25% had an SLNB, with 0% positive rate. The follow-up range was 2 years, 3 months–10 years, 8 months, with median of 2 years, 8 months and a mean of 5 years, 2 months. A proportion of 75% were alive without disease at the time of the study and 25% were LTFU.

For the atypical cellular blue nevus, the lesion was on the hand of a male patient diagnosed at age 15 and was treated with WLE. The patient is alive without disease with a 7-year, 4-month follow-up duration.

## 4. Discussion

### 4.1. Melanomas

Pediatric cutaneous melanomas impact an estimated 1–11 per 1 million children, and comprise 3% of all pediatric cancer diagnoses [5,6,10,11]. Our study identified 53 patients diagnosed with malignant melanoma at a single institution over twenty years (Table 1). The median age at diagnosis was 16 years, consistent with the current literature, suggesting that pediatric melanoma diagnoses are more prevalent in adolescents than in children [5,10,26]. One notable exception to this trend was seen in a 20-year, 37-case cohort that showed a median age of 10 for cases of pediatric melanoma in Australia and New Zealand [3]. There was a slightly higher percentage of female patients with melanoma at 51%, consistent with studies that suggest females have a higher [15] or comparable [3] incidence of pediatric melanoma than males. For the location of disease, over half of the cases were melanoma of the trunk, similar to other data suggesting the trunk is a common primary location for cutaneous pediatric melanoma [5,7], or the highest incidence in patients of adolescent age group [4,15]; however, studies have shown that the extremities [5] and head and neck [3] were the most common.

For treatment methodology, 96% of patients underwent WLE, which is consistent with the standard of care for adults, although there are no consistent guidelines for the pediatric population [4]. In addition, most patients described in this study underwent SLNB if Breslow thickness was greater than 1 mm (95%, 21/22), which is the American Joint Commission on Cancer (AJCC) standard care for adults; further studies are necessary to determine if whether this should be standard for pediatric patients [6]. Many who underwent SLNB had positive results (35%), higher than the 25% positive rate reported in a previous study (for children < 20 years old) [7], but comparable to the more recent cohort study in Australia and New Zealand oncology centers which reported 38% positive rate (children 0–18 years old) [3]. This higher SLNB rate may, in part, be due to our study location, which is a regional referral center, whereas a lower positive rate was reported using SEERs data [7].

Although a larger portion of the patients had positive SLNB than previously described, 75% were alive at the time of study without recurrence. This survival rate is comparable to, but lower than, the 85% survival rate described by Dean et al. [5] in their 35-year cohort study in British Columbia, less than the reported 87–95% overall 5-year survival rate in Saiyed et al. review article [4], but higher than the 67.7% overall survival reported by Ryan et al. in their 20-year cohort study in Australia and New Zealand [3]. It is noteworthy that the British Columbia study examined all cases in the province, where this study and that in Australia and New Zealand examined cases at a tertiary care and cancer center.

It is unclear how much Breslow thickness influenced outcomes. Shi et al. [10] found no significant difference in outcomes between thicknesses in pediatric patients ages 0–21 years old. The average Breslow thickness for this study was 1.68 mm, smaller than that found in Shi et al. (2.13 mm), although this may not be a good measure of outcomes. From our cohort, those with metastatic disease had a Breslow thickness that was much higher (average 5.23 mm) than the overall average for melanoma; this is consistent with the known prognostics of Breslow thickness for conventional melanoma in the adult population. Further data are necessary to determine how Breslow thickness impacts the treatment and prognosis in the pediatric melanoma population [10].

Four patients died from disease. One had a primary lesion with a Breslow thickness of 5 mm. Their disease had distant metastasis to the right occipital lymph node, brain, lungs, and bone. Their chemotherapy regimen included the following (many transitions due to side effects): interferon, ipilimumab/nivolumab, infliximab, dabrafenib, and trametinib. The lesion tested positive for BRAF V600E, but negative for KIT or NRAS mutations. Another patient had a primary lesion with a 13 mm Breslow thickness. Metastatic sites included epidural, left axillary nodes, mediastinal lymph nodes, and bone. The lesion also tested positive for BRAF V600E. This patient was treated with ipilimumab and then dabrafenib. The third patient had a Breslow thickness of 1.02, with positive SLNB and metastatic lesions in the chest wall, lung, abdominal cavity, peritoneal cavity, femur and pelvis, spine, ventricular ependymal lesions, leptomeningeal, and numerous cerebral, cerebellar, and soft tissue lesions. This patient had WT BRAF and received ipilimumab, nivolumab, and vemurafenib for systemic chemotherapy. It is noteworthy that this patient had a history of intracranial PNET (Ewing Sarcoma), and the melanoma was in the radiation field. The final patient who died of disease was a patient with a malignant melanoma of the spindle cell type arising in the background of a large congenital lesion. The Breslow thickness was not assessed, and this patient received WLE but not SLNB was documented. There were multiple recurrences of the lesion documented, but there are no data on distant metastases or systemic chemotherapy and the patient died of disease.

Only 13% (7/55) of patients underwent BRAF testing in this cohort, comparable to recent cohort study with 18% tested for BRAF [3]. Of those, 71% (5/7) tested for positive BRAFV600E mutation which is present in melanoma of both adult and pediatric populations, much higher than the 14% described in that same cohort study [3]. A proportion of 80% (4/5) of the patients whose lesion tested positive for BRAF V600E received anti-BRAF therapy in our cohort (one patient did not have systemic metastatic disease and did not receive any systemic chemotherapy). Only 40% (2/5) of the patients in this cohort with BRAF V600E mutations were alive without disease at the time of this study, and 60% (3/5) were deceased with disease. This study provides seven additional examples of patients with pediatric melanoma who underwent BRAF testing, yet more research is necessary to determine the influence of BRAF mutations on pediatric melanoma outcomes.

Overall, our cohort displayed characteristics that were consistent with the literature (age at diagnosis, WLE rate, survival rate), with some unique features (higher SLNB-positive rate).

### 4.2. Atypical Spitz Proliferations

In atypical Spitz proliferations, the median age at diagnosis was 7 years old for patients with all atypical Spitz tumor types (*n* = 101) (Table 2), which is consistent with the literature, reporting an average age of 7.5 years [2]. The most common location for these lesions was on the lower extremities, which is also consistent with the literature [19,20]. Our cohort was 47% male, 53% female; prior literature reported equal sex distribution [2].

Currently, guidelines suggest WLE of atypical Spitz tumors, but there is no clear evidence to show that performing SLNB adds a clinical benefit [2]. In our cohort, the percentage of patients that underwent WLE was 77–19%; these patients also received an SLNB. Massi et al. suggests all atypical Spitz tumors should be excised given their differences histologically [2]. That study also suggests there may be no clinical benefit to SLNB, given its low rate of metastases. Other studies concur that, even with a positive SLNB from an atypical Spitz tumor, it does not share same negative diagnostic outcome as a positive SLNB would have in the case of malignant melanoma [27,28]. In our series, 15 patients underwent sentinel lymph node biopsy, of which 3 were positive for lymph node disease. Systemic metastasis was seen in one case. Almost three quarters of the patients were alive without disease at the time of this study, although about 20% of patients were LTFU, consistent with the favorable outcomes suggested for these entities [2,17,28]. Given the outcomes in this cohort, there may not be utility of an SLNB in atypical Spiz tumors (in the absence of histological or clinical concern for metastasis), as WLE provides sufficient treatment and positive long-term outcomes for these patients.

Of the 101 atypical Spitz proliferations, 15% were Spitz melanomas. The near 50/50 split in sex at diagnosis was consistent with the literature for Spitz-type melanomas [29]. The extremities (6/15) were the most common location, consistent with the literature [20,29,30]. The mean age of 10.5 years was near the previously reported mean of 9.9 years in a 38-patient cohort [29]. A series of 56 patients with atypical Spitz tumors and melanomas in the pediatric and adult population found a median Breslow thickness of 2.85 mm in 52 cases with favorable behavior and 5.25 mm in those with unfavorable behavior [19]. A second series of 38 pediatric (less than 18) Spitz melanomas found an average Breslow of 3 mm [29]. In our cohort of 15, the average Breslow thickness was 2.54 mm. As for treatment, 93% (14/15) of patients received a WLE, the current standard of care [2,20,28], but the one patient who did not receive WLE was recommended to have this procedure. A proportion of 80% of patients had an SLNB (12/15), with a 25% (3/12) positive rate, which has questionable predictive value for prognosis according to the literature [2,17,27,28].

One patient did die of disease. The pathologic diagnosis was that of Spitz melanoma. The patient had systemic metastasis to the following locations: regional lymph node, a 3 cm extra-nodal mass, and CT showing lung masses suspicious for lung metastases. This patient did not receive immunotherapy; ipilimumab was discussed but not initiated. Typically, Spitz melanoma is viewed as having more favorable outcomes than malignant melanoma of other subtypes; however, there are a rare subset of Spitz melanomas that can behave aggressively [19,27,31]. It is noteworthy that the patient had a lesion removed three years prior to Spitz melanoma diagnosis that was called an atypical Spitz nevus with negative margins. Molecular analysis on this case showed WT BRAF; there were no additional testing performed on this case, so it is unknown whether this patient’s disease harbored the TERT mutation that has been suggested to confer a worse outcome [19]. There have been documented cases of fatal Spitz melanoma [2,20,29]. Given that one patient out of 101 cases died of disease, the mortality rate is consistent with the reported percentage of less than 5% for atypical Spitz tumors [2]. That said, 6.7% of the cases of Spitz melanoma were fatal in this cohort, highlighting that this histologically distinct and concerning entity can be fatal. Consistent with the literature, the cohort described in this paper had atypical Spitz tumors and melanoma most commonly of the lower extremities, appearing in preadolescent children.

### 4.3. Atypical Melanocytic Tumors, Not Otherwise Specified

Of the cases that did not fall into the listed categories above, we had five cases of pigmented epithelioid melanocytoma (PEM) and four atypical deep-penetrating nevi, both of which are exceedingly rare (Table 3). We also had a case of atypical cellular blue nevus.

PEM typically occurs as a solitary tumor in healthy children or adults and has a high propensity (46%) [24] to spread to regional lymph nodes; the cohort described in this paper had 50% positive sentinel lymph biopsy rate, and one case of regional spread. None of the patients had widely metastatic disease, consistent with the literature suggesting that it is uncommon [23]. The PEM case which had a positive lymph node and regional lymph node spread, was treated with interferon. All cases were treated with WLE. A proportion of 100% of the patients were alive without disease at the time of the study without evidence of recurrence, consistent with the limited literature [23,24].

Atypical deep-penetrating nevi can occur in children and adults and has limited literature and a difficult histopathologic diagnosis [1]. In a systematic review of adult and pediatric cases [1], a higher incidence in females was seen, with the most common location of disease being the head and neck, followed by the extremities. Our cohort of four had 75% male cases, in contrast to this study, but aligns with Gil et al. summarized variable sex distributions in various cohort studies, although this reviewed adult and pediatric patients [25]. For this small cohort, 50% of cases were of the head and neck, and 50% were of the trunk. Excision was the most common treatment modality seen in Cosagarea et al. 2020 [1] systematic review of 355 DPN and 48 cases of borderline DPN, and in the case of atypia, WLE is the suggested standard of care [25]; this is consistent with this small cohort (50% WLE rate). One patient had a negative SLNB, which is consistent with the literature showing low SNLB rate, low SNLB positivity, and few recurrences of metastatic disease seen with long term follow-up [1,25]. The majority of these patients were alive without disease at the time of study, with one patient lost to follow-up.

Wide local excision may be the definitive treatment for these rare melanocytomas; however, it is still unclear whether performing SLNB is necessary. For these rare entities, the lack of recurrence and 100% survival rate are consistent with the limited literature.

### 4.4. Limitations

There were many limitations to our study, including data pulled from a single institution with relatively small patient cohorts identified. Additionally, some patients were LTFU making studying follow-up times challenging to collect and interpret. Data were not stratified for socially defined race, and some data were not stratified for tumor stage, both of which could present bias to the data. In general, more research with larger cohorts from multiple locations would be beneficial to study long-term outcomes, although given the rare nature of these entities, may be challenging.

## 5. Conclusions

Given the limited literature on pediatric melanomas, atypical Spitz tumors, and other rare melanocytomas, we presented additional data consistent with features and characteristics as those in the literature, such as survival rates, location of disease, and overall standard-of-care treatment rates. There were distinct exceptions such as a higher melanoma SLNB-positive rate, which may be due in part from the institutional patient population in this study. The lower WLE rate with data suggesting positive prognosis may suggest that, in the absence of concerning features of melanoma, these atypical Spitz proliferations may not require WLE, but more data are necessary to determine optimal treatment for these patients. Additionally, our study highlights a case of metastatic and fatal Spitz melanoma, a rare outcome for patients with this condition. By providing more data on these proliferations, insight in disease course and outcomes for these patients and their families can be elucidated.

## Figures and Tables

**Table 1:** Summary of pediatric melanoma cases.

Summary of Melanoma Cases	
Number of patients	53
Number of MM cases total	55
Age range at Dx	4 months–18 years
Median age at Dx (mean)	16 (14 years, 11 months)
% M, % F	49% M, 51% F (26M/27F)
Location	Trunk (53%, 29), head and neck (22%, 12), legs (15%, 8), arms/hands (5%, 3), feet (5%, 3)
Average Breslow thickness (range)	1.68 (0.2–13) mm
% WLE	96% (53/55; 1 scheduled for WLE, other biopsy slide consult only)
% SLN biopsy	47% (26/55)
% Positive SLN	35% (9/26)
% Systemic Met disease	7% (4/55)
Average Breslow thickness for patients with systemic Met disease (median)	5.23 (3.45) mm
% Alive without disease	75% (40/53)
% LTFU	15% (8/53)
% Deceased with disease	8% (4/53)
% Systemic chemotherapy	17% (9/53)
Median Follow-up time (mean) overall	8 years, 11 months (9 years, 2 months)
BRAF mutation	5 patients

**Table 2:** Summary of pediatric atypical Spitz tumor (AST) cases.

Summary of AST Cases	
Number of patients	101
% M, % F	47% M, 53% F (47M/54F)
Age range at diagnosis	7 months to 18 years
Median (mean) age at diagnosis overall	7 years (8 years, 5 months)
Location	Legs (30%, 30), arms/hands (25%, 25), head and neck (24%, 24), trunk (18%, 18), feet (4%, 4)
% WLE	77% (78/101)
% SLNB? (%WLE + SLNB)	19% (15/78)
% Positive SLNB	20% (3/15)
% with Metastatic disease	1% (1/101)
Systemic chemo	0%
Follow-up time	Between 6 month and 19 years, 11 months
Median (mean) overall follow-up time	9 years (8 years 10 months)
Median (mean) follow-up for Spitz melanoma	8 years 7 month (8 years 6 months)
% Alive without disease	74% (75/101)
% LTFU	20% (20/101)
% Alive with disease/unknown if underwent further excision	5% (5/101)
% deceased with disease	1% (1/101)
% Breakdown of AST types	15% (15) melanoma (called or treated as such), 85% (86) AST
Average Breslow Thickness for Spitz Melanoma (range)	2.54 mm (0.6–5.4 mm)

**Table 3:** Summary of atypical melanocytic proliferations NOS cases.

	Summary of Atypical Melanocytic Tumors NOS Cases	
PEM	Number of patients	5
	Age range at Dx	3 to 16 years
	Median (mean) age at Dx	10 (9 years, 7 months)
	% M, % F	80% M, 20% F
	Location	Head and neck (2), trunk (2), leg (1)
	% WLE	100%
	% SNLB	80%
	% positive SNLB	50% (2/4)
	% with Metastatic disease	1%
	% Alive without disease	100%
	% Systemic chemotherapy	20% (1/5, interferon)
	Follow-up time range (median, mean)	3 years, 8 months to 12 years, 8 months (6 years, 11 months, 8 years, 2 months)
Deep-penetrating Nevi		
	Number of patients	4
	Age range at Dx	2 to 18 years
	Median (mean) age at Dx	14 years 6 months (12 year, 3 months)
	% M, % F	75% M, 25% F
	Location	Head and neck (2), trunk (2)
	% WLE	50%
	% SNLB	25%
	% positive SNLB	0%
	% with Metastatic disease	0%
	% Alive without disease	75%
	% Systemic chemotherapy	25%
	Follow-up time range (median, mean)	2 years, 3 months to 10 years, 8 months (2 years, 8 months, 5 years, 2 months)

## Data Availability

Data are available on request due to restrictions (privacy).
